# 

**Published:** 1895-02

**Authors:** 


### CONTENTS:

### SELECTIONS.

Article I.—A New Era in Dental Practice. By Prof. Flagg. - 433
" II.—Filling Cervical Cavities. - - - - - - - - 451
" III.—Percelain Work. J. H. Downie, D. D. S. - - - - 453
" IV.—A Pathological Condition of the Mucous Glands of the Mouth. By Geo. Howe Winkler, M. D., D.D.S. 459

### EDITORIAL, ETC.

An Ideal Business and Social Meeting of Dentists. - - - - - 469
Disinfection of Instruments. - - - - - - - - - - - 469

### OBITUARY.

Dr. William H. Hoopes. - - - - - - - - - - - - - - 470

### MONTHLY SUMMARY.

Failures to be Avoided. - - - - - - - - - - - - - - 470

Filling Roots. - - - - - - - - - - - - - - - 471

Tin and Amalgam Filling. - - - - - - - - - - - 472

Making Steel Crown Dies. - - - - - - - - - - - 473

Bleaching Teeth. - - - - - - - - - - - - - - 473

Coagulants. - - - - - - - - - - - - - - - - 474

Sawdust Bread. - - - - - - - - - - - - - - - 474

Teething Powders. - - - - - - - - - - - - - - 475

Coffee Kills Germs. - - - - - - - - - - - - - - 475

The Parts that do not Grow Old. - - - - - - - - - 476

The Effects of Intense Cold upon the Mind. - - - - - - 476

Dry Surgery in Germany. - - - - - - - - - - - - 477

Painless Extraction Thirty Years Ago. - - - - - - - - 478

Fast Living. - - - - - - - - - - - - - - - - 478

What Hot Springs and Hot Baths Will Do. - - - - - - - 479

A Survival. - - - - - - - - - - - - - - - - - 479

Death is Painless. - - - - - - - - - - - - - - 480

